# Defects, Diffusion, and Dopants in Li_2_Ti_6_O_13_: Atomistic Simulation Study

**DOI:** 10.3390/ma12182851

**Published:** 2019-09-04

**Authors:** Navaratnarajah Kuganathan, Sashikesh Ganeshalingam, Alexander Chroneos

**Affiliations:** 1 Department of Materials, Imperial College London, London SW7 2AZ, UK; 2 Faculty of Engineering, Environment and Computing, Coventry University, Coventry CV1 5FB, UK; 3 Department of Chemistry, University of Jaffna, Sir. Pon Ramanathan Road, Thirunelvely, Jaffna 40000, Sri Lanka

**Keywords:** Li_2_Ti_6_O_13_, defects, Li-ion diffusion, dopants, atomistic simulation, DFT

## Abstract

In this study, force field-based simulations are employed to examine the defects in Li-ion diffusion pathways together with activation energies and a solution of dopants in Li_2_Ti_6_O_13_. The lowest defect energy process is found to be the Li Frenkel (0.66 eV/defect), inferring that this defect process is most likely to occur. This study further identifies that cation exchange (Li–Ti) disorder is the second lowest defect energy process. Long-range diffusion of Li-ion is observed in the *bc*-plane with activation energy of 0.25 eV, inferring that Li ions move fast in this material. The most promising trivalent dopant at the Ti site is Co^3+^, which would create more Li interstitials in the lattice required for high capacity. The favorable isovalent dopant is the Ge^4+^ at the Ti site, which may alter the mechanical property of this material. The electronic structures of the favorable dopants are analyzed using density functional theory (DFT) calculations.

## 1. Introduction

Energy demand arising from non-renewable fossil fuels has led to research activity towards alternative renewable energy devices. Rechargeable batteries have the potential to store clean energy without the hazards of carbon emission.

Rechargeable lithium-ion batteries (LIBs) are promising in portable energy storage applications due to the low weight and non-toxicity of lithium. Producing an efficient LIB requires promising electrode and electrolyte materials. A variety of materials have been examined for the last two decades to make LIBs promising [1,2,3,4,5,6,7,8,9,10,11,12].

Lithium titanates, such as Li_2_Ti_3_O_7_, LiTi_2_O_4_, and Li_4_Ti_5_O_12_, were recently considered as anode materials for LIBs due to their zero-strain behavior upon Li intercalation/de-intercalation, though they exhibit low ionic and electronic conductivity [13,14,15]. The long cycle life of such materials is due to the structural stability arising from zero strain and would lead to the development of LIBs with long life. Spinel-type Li_4_Ti_5_O_12_ is an attractive anode material because of its reversible Li intercalation/de-intercalation at ~1.6 V [15,16]. Furthermore, Li_2_Ti_3_O_7_ has been identified as a fast Li-ion conductor [17].

Lithium hexatitanate (Li_2_Ti_6_O_13_) is another type of titanate exhibiting one-dimensional tunnels in the crystal structure needed for fast Li-ion diffusion [18]. Li_2_Ti_6_O_13_ has been synthesized and its electrochemical properties examined [18,19,20]. Kataoka et al. [18] used an ion exchange experiment to prepare Li_2_Ti_6_O_13_ from Na_2_Ti_6_O_13_. Their study showed that a stable, reversible capacity of ~90–95 mA·h·g^−1^ was observed after the first cycle. Furthermore, conductivity measurements show that this material exhibits a good Li-ion conduction at room temperature. In a different experimental study [19], it was shown that a five Li per formula unit can be inserted into Li_2_Ti_6_O_13_ between 1.5 and 1.0 V. Density functional theory (DFT) and a classical simulation study performed by Zulueta et al. [21] showed that Li_2_Ti_6_O_13_ is a semiconductor, having a band gap of 3.10 eV, and the activation energy for the Li-ion diffusion is 0.47 eV, though the Li-ion diffusion path was not reported. 

The present study aims to examine the crystallographic defects in Li-ion diffusion paths together with activation energies and the impact of dopants in these materials, using force field methods, as reported in previous studies [22,23,24,25,26,27,28,29,30,31,32,33,34,35,36,37,38,39,40,41,42,43,44] for various battery materials. DFT method was applied to study the electronic structure of promising dopants substituted at the Ti site in Li_2_Ti_6_O_13_.

## 2. Computational Methods

The General Utility Lattice Program (GULP Version 3.4.7) [45] was used to run atomistic simulations based on the classical force field. In this simulation code, lattice energy is calculated by considering Coulombic (long-range) attraction and short-range repulsion (i.e., Pauli electron-electron) and attraction (i.e., van der Waals). To model short-range interaction, Buckingham potentials [see Table S1 in the Supplemental Materials] were used. The Broyden–Fletcher–Goldfarb–Shanno (BFGS) algorithm [46] as implemented in the GULP code was used to model perfect and defect structures of Li_2_Ti_6_O_13_. In all-relaxed configuration, the forces on the atoms were less than below 0.001 eV/Å. Point defects were modeled using the Mott–Littleton method [47]. An overestimation in the defect enthalpies was expected due to the low concentration of ions with spherical shape. Nevertheless, the relative energy trend would be consistent. In the current simulation, isobaric parameters were used to calculate the formation and migration energies. In previous work, we have discussed the thermodynamical relations associated with isobaric parameters in detail [48,49,50,51,52]. 

Spin-polarized DFT calculations, as implemented in the Vienna Ab initio Simulation Package (VASP Version 5.3.5) [53,54], were performed to examine the electronic structures of the promising dopants substituted at the Ti site. The generalized gradient approximation (GGA) as described by Perdew, Burke, and Ernzerhof (PBE) [55] was used to model the exchange-correlation term. A plane-wave basis set with a cut off value of 500 eV was used. Defect modeling was performed in a supercell containing 126 atoms. In all cases, a 2 × 2 × 2 Monkhorst–Pack *k*-point mesh [56] containing 8 *k* points was used. Geometry optimizations were performed using a conjugate gradient algorithm [57]. Using the Hellman–Feynman theorem together with Pulay corrections, forces on the atoms were obtained. Forces on the atoms were smaller than 0.001 eV/Å, and the stress tensor was less than 0.002 GPa in all optimized configurations. Dispersion was applied in all calculations in the form of a pair-wise force field as parameterized by Grimme et al. [58] (DFT-D3) in VASP.

## 3. Results and Discussion

### 3.1. Crystal Structure of Li_2_Ti_6_O_13_

**Figure 1:**
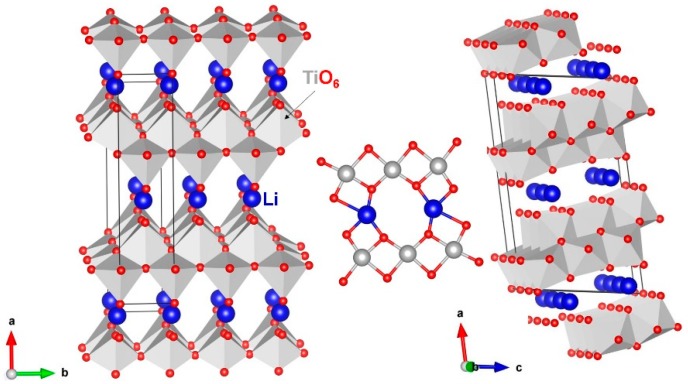
Monoclinic crystal structure of Li_2_Ti_6_O_13_ (space group C2/m).

Li_2_Ti_6_O_13_ crystalizes in the monoclinic system (space group C2/m). Figure 1 shows the crystal structure of Li_2_Ti_6_O_13_. Kataoka et al. [18] synthesized monoclinic Li_2_Ti_6_O_13_ using sodium/lithium ion exchange method from monoclinic Na_2_TiO_3_. Lattice parameters from their study were reported to be a = 15.3065 Å, b = 3.74739 Å, c = 9.1404 Å, α = γ = 90.0°, and β = 99.379°. In the crystal structure, Ti forms distorted octahedra, and they were interconnected by edge sharing. A distorted planar LiO_4_ unit was observed, and this planar coordination is not normal for Lithium.

The validity of the Buckingham potentials used in the force field method and the projector augmented wave (PAW) potentials [59] used in the DFT method were tested by performing a full geometry optimization of bulk Li_2_Ti_6_O_13_ under constant pressure. The calculated structural parameters are in good agreement with the experimental values reported by Kataoka et al. [18]. The calculated and experimental lattice parameters and angles are listed in Table 1.

### 3.2. Intrinsic Defect Process

**Figure 2:**
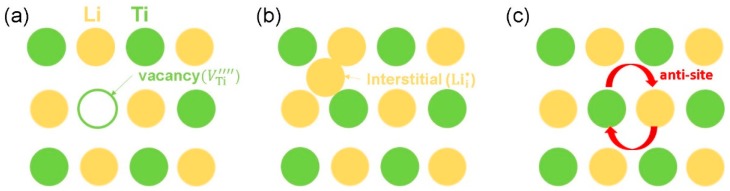
Schematic diagrams showing (**a**) vacancy, (**b**) interstitial, and (**c**) anti-site defects in Li_2_Ti_6_O_13_.

Defects in a material are important, as they influence the properties’ materials in many ways. Diffusion is one of the important properties that is dominated by defects. Anti-site defects can change the mechanical property of the material and the concentration of the point defects. Current atomistic simulation method enabled us to examine the defects. Schematics showing vacancy, interstitial, and anti-site defects are reported in Figure 2. In this section, we examine the process of calculating the Schottky, Frenkel, and anti-site defect energies. First, we calculated the formation energies of point defects (vacancies and interstitials), and then resulting energies were used to evaluate the Schottky and Frenkel defect formation energies. The Li–Ti anti-site defect process was considered by exchanging their positions. The intrinsic point defects are important, as they influence the ion diffusion in the crystal. We used Kröger–Vink notation [60] to represent the Schottky, Frenkel, and anti-site reaction energy processes derived by combining the point defects. The reaction equations decribing the defect processess are as follows:

(1) $$\mathrm{Li}\;\mathrm{Frenkel}:{\;\mathrm{Li}}_{\mathrm{Li}}^{X}\;\to \;V_{\mathrm{Li}}^{\prime }+{\;\mathrm{Li}}_{i}^{•}$$

(2) $$O\;\mathrm{Frenkel}:{\;O}_{O}^{X}\;\to \;V_{O}^{••}+{\;O}_{i}^{\prime \prime }$$

(3) $$\mathrm{Ti}\;\mathrm{Frenkel}:{\;\mathrm{Ti}}_{\mathrm{Ti}}^{X}\;\to \;V_{\mathrm{Ti}}^{\prime \prime \prime \prime }+{\;\mathrm{Ti}}_{i}^{••••}$$

(4) $$\mathrm{Schottky}:2{\;\mathrm{Li}}_{\mathrm{Li}\;}^{X}+\;6{\mathrm{Ti}}_{\mathrm{Ti}}^{X\;}+\;13{\;O}_{O}^{X}\to \;2\;V_{\mathrm{Li}}^{\prime }+\;6\;V_{\mathrm{Ti}}^{\prime \prime \prime \prime }\;+\;13\;V_{O}^{••}+{\mathrm{Li}}_{2}{\mathrm{Ti}}_{6}O_{13}$$

(5) $${\mathrm{Li}}_{2}O\;\mathrm{Schottky}:2{\;\mathrm{Li}}_{\mathrm{Li}}^{X}+{\;O}_{O}^{X\;}\to 2\;V_{\mathrm{Li}}^{\prime }+V_{O}^{••}+{\;\mathrm{Li}}_{2}O$$

(6) $${\mathrm{TiO}}_{2}\;\mathrm{Schottky}:{\;\mathrm{Ti}}_{\mathrm{Ti}}^{X}+\;2{\;O}_{O}^{X\;}\to \;V_{\mathrm{Ti}}^{\prime \prime \prime \prime }+2\;V_{O}^{••}+{\;\mathrm{TiO}}_{2}$$

(7) $$\mathrm{Li}/\mathrm{Ti}\;\mathrm{antisite}\;\left(\mathrm{isolated}\right):{\;\mathrm{Li}}_{\mathrm{Li}}^{X}+{\;\mathrm{Ti}}_{\mathrm{Ti}}^{X\;}\to {\mathrm{Li}}_{\mathrm{Ti}}^{\prime \prime \prime }+{\mathrm{Ti}}_{\mathrm{Li}}^{•••}$$

(8) $$\mathrm{Li}/\mathrm{Ti}\;\mathrm{antisite}\;\left(\mathrm{cluster}\right):{\;\mathrm{Li}}_{\mathrm{Li}}^{X}+{\;\mathrm{Ti}}_{\mathrm{Ti}}^{X}\to \;\{{\mathrm{Li}}_{\mathrm{Ti}}^{\prime \prime \prime }:{\mathrm{Ti}}_{\mathrm{Li}}^{•••}\text{\}}.$$

**Figure 3:**
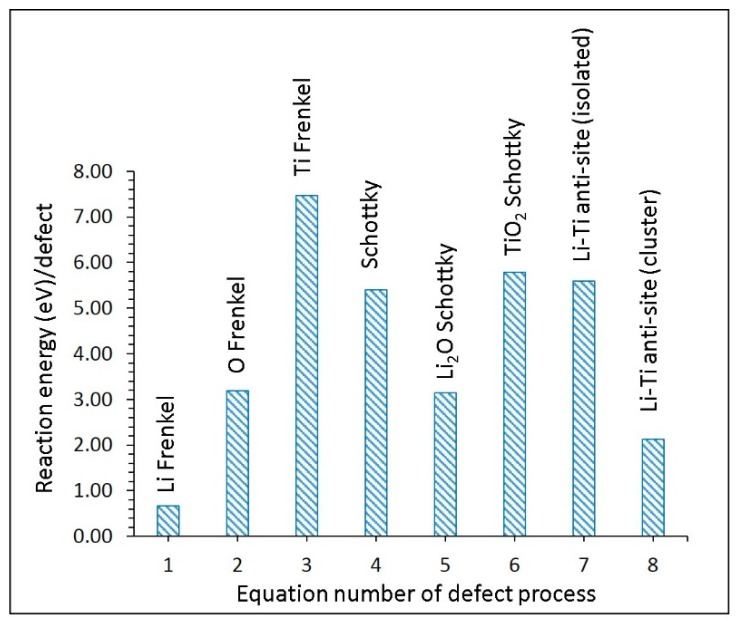
Energetics of intrinsic defect processes.

Figure 3 shows the energy per defect for each of the defect processes. The lowest energy was for the Li Frenkel (0.66 eV/defect). This defect process can facilitate the formation of Li vacancies required for the Li-ion diffusion in Li_2_Ti_6_O_13_. The second most favorable defect was the Li–Ti anti-site defect cluster (2.13 eV/defect), but its magnitude was high, implying that a small amount of such defect can be present only at high temperatures. In previous experimental and theoretical studies of oxide materials, this defect has been discussed [6,24,61,62,63,64]. The formation energy of Li_2_O was calculated to be 3.14 eV/defect, suggesting that the loss of Li_2_O in this material requires high temperatures. Schottky and other Frenkel defect energies were high, suggesting that such defect processes are not significant in this material.

### 3.3. Lithium-Ion Diffusion

**Figure 4:**
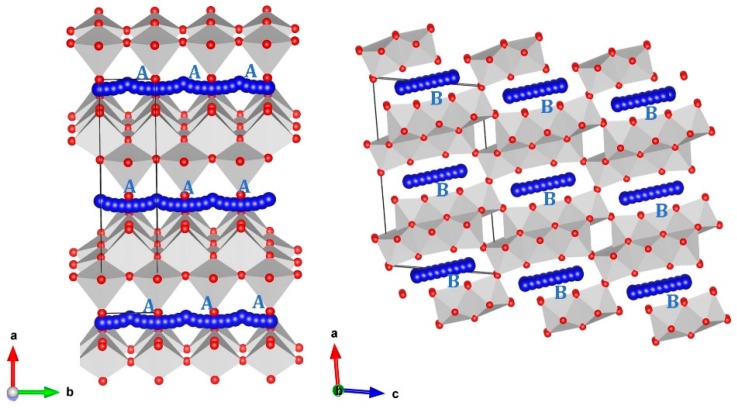
Li-ion diffusion paths calculated in Li_2_Ti_6_O_13_. Blue atoms correspond to local Li-ion hopping trajectories.

**Figure 5:**
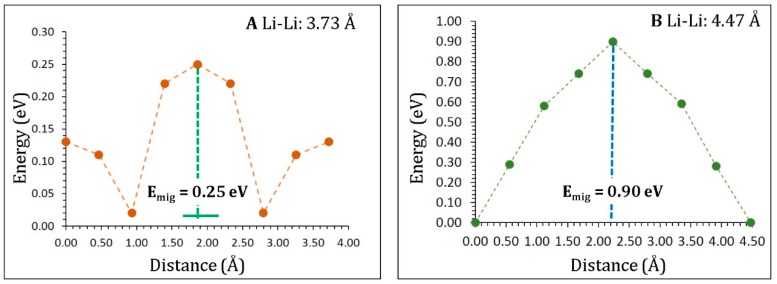
Energy profile diagrams for Li-ion local hops **A** and **B** as shown in Figure 4.

A promising electrode material requires high Li-ion diffusion. The present force field-based simulation enables the calculation of long-range Li-ion diffusion pathways with activation energies. Two different local Li hops, A and B (see Figure 4), were identified with jump distances of 3.73 Å and 4.74 Å, respectively. Activation energies together with the Li–Li separations are listed in Table 2. Figure 5 shows the energy profile diagrams for the local Li hops (A and B). Diffusion of Li ions in hop A can be observed in the *bc*-plane with an activation energy of 0.25 eV, implying very fast Li-ion diffusion in agreement with the experimental measurements reported by Kataoka et al. [18]. Li ions migrated in hop B with a significantly higher activation energy of 0.90 eV. This was due to the longer jump distance than that observed in hop A.

Next, we constructed possible long-range Li-ion diffusion pathways by connecting local hops. Only one possible long-range Li-ion diffusion channel consisting of local hops A was identified. A long-range movement of Li-ion along the *b* direction in the *bc*-plane was observed. The activation energy was 0.25 eV for this long-range diffusion. Local hops B could not form long-range diffusion, as these hops were discontinuous in the lattice. The calculated activated energy was lower than the value calculated for other titanates, such as Li_2_TiO_3_ (0.51 eV) [34] and Li_2_Ti_3_O_7_ (0.67–0.74 eV) [65]. The Li-ion diffusion barrier in Li_2_Ti_6_O_13_ was reported to be 0.47 eV by Zulueta et al. [21]. Nevertheless, the direction of diffusion was not reported. In the present study, we calculated both the migration pathways and their activation energies.

In line with previous studies [22,23,24,25,26,27], Li-ion migration calculations were performed by following the method developed by Catlow et al. [66]. Two adjacent Li vacancy sites were first created, and Li-ion interstitial positions were then systematically placed at regular intervals along the diagonal connecting them. Seven interstitial positions were considered in all cases and the interstitial ion was fixed while all other ions were free to relax. However, fixing the interstitial ion position does not guarantee the minimum energy path, and it will give only a direct diffusion path. Therefore, interstitial positions were allowed to move x, y, z, xy, yz, and xz directions separately. Finally, the lowest activation energy pathway (curved pathway) was reported. The difference in energy between the saddle point position and the system in its initial state was calculated and reported as the activation energy.

### 3.4. Trivalent Doping

Generating extra lithium in Li_2_Ti_6_O_13_ can increase its capacity. A way to achieve this is by doping trivalent dopants at the Ti site, as this process can instigate Li interstitials in the lattice according to the following reaction:

(9) $$R_{2}O_{3}+2{\mathrm{Ti}}_{\mathrm{Ti}}^{X}+{\;\mathrm{Li}}_{2}O\to \;2{\;R}_{\mathrm{Ti}}^{\prime }+2{\;\mathrm{Li}}_{i}^{•}+\;2\mathrm{Ti}O_{2}.$$

**Figure 6:**
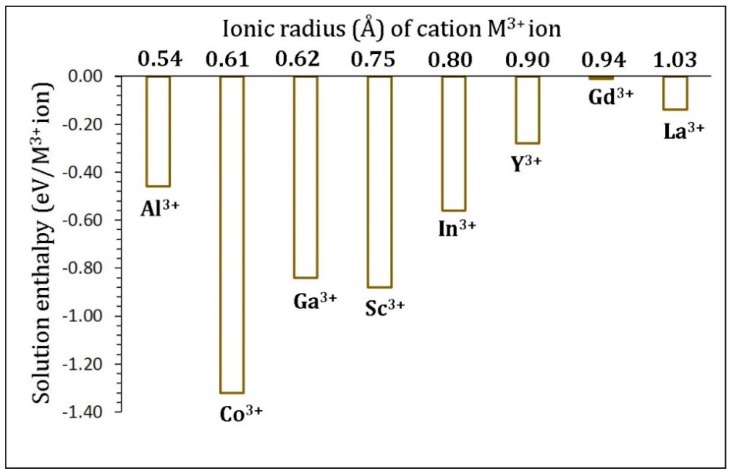
Solution enthalpies calculated for R_2_O_3_ (R = Al, Co, Ga, Sc, In, Y, Gd, and La) with reference to the M^3+^ radius in an octahedral coordination.

Figure 6 shows the solution enthalpies calculated for this process. In all cases, solution enthalpies are exothermic, meaning that they are all candidate dopants for this process. The most promising dopant is Co^3+^ with the solution enthalpy of −1.32 eV. The least favorable dopant is Gd^3+^. In a previous experimental study [67], substitutional doping by Co^3+^ at the Ru site was performed in Li_2_RuO_3,_ and the resultant over-lithiated Li_2+x_Ru_1−x_CoO_3_ compound exhibited an improvement in the electrochemical lithium reversibility and extraction of the Li^+^ ion compared with the un-doped Li_2_RuO_3_. In general, there is a reduction in the solution enthalpy with ionic radius from Co to La. 

### 3.5. Tetravalent Doping

The isovalent doping process by displacing Ti with Si, Ge, Mn, Sn, and Ce is considered here. This doping strategy can alter the mechanical, electrical, and optical properties of Li_2_Ti_6_O_13_. Solution enthalpy for this process was calculated using the following equation:

(10) $$RO_{2}+{\mathrm{Ti}}_{\mathrm{Ti}}^{X}\;\to \;R_{\mathrm{Ti}}^{X}+\mathrm{Ti}O_{2}.$$

**Figure 7:**
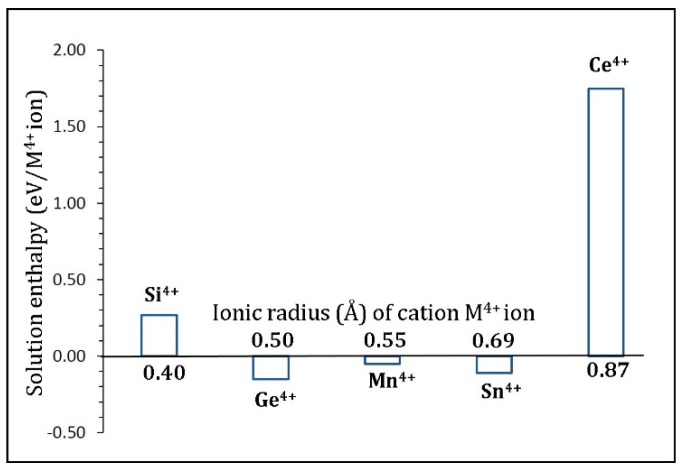
Solution enthalpies calculated for RO_2_ (R = Si, Ge, Mn, Sn, and Ce) at the Ti site with reference to M^4+^ radius in an octahedral coordination.

Figure 7 reports the solution enthalpies. Exoergic solution enthalpies are noted for Ge^4+^, Mn^4+^, and Sn^4+^, while dopants Si^4+^ and Ce^4+^ exhibited endoergic solution enthalpies. The most favorable promising dopant is Ge^4+^. Both Mn^4+^ and Sn^4+^ are also worth examining experimentally. The solution enthalpy for Ce^4+^ is 1.75 eV, suggesting that a high temperature is needed for this dopant. In a theoretical study by Zulueta et al. [21], it was predicted that Li_2_Sn_6_O_13_ can be synthesized by Ti^4+^/Sn^4+^ ion exchange method. This supports our exoergic solution enthalpy for the doping of Sn^4+^ at the Ti site. Future experimental studies need to consider the exchange of Ti with Ge^4+^ and Mn^4+^, as these two dopants also exhibit exoergic solution enthalpy.

Substitutional doping can impact the Li-ion diffusion barrier because of the change in the local environment. In our recent study [43], we showed that the activation energy of Na-ion diffusion slightly changes upon doping due to reduction or elongation in the Na–Na distance in NaNiO_2_. 

### 3.6. Electronic Structures of Co^3+^- and Ge^4+^-Doped Li_2_Ti_6_O_13_

**Figure 8:**
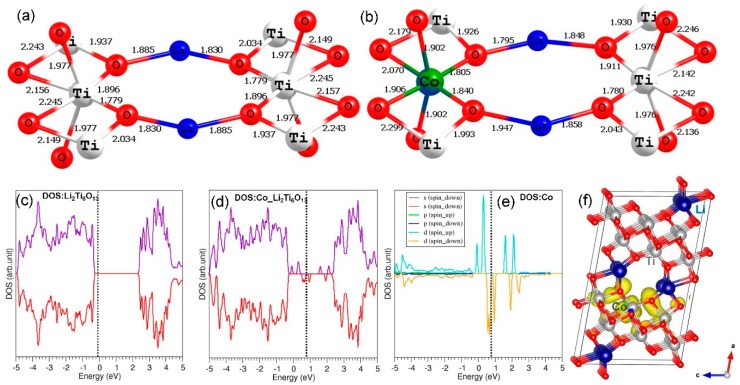
(**a**) TiO_6_ octahedral unit in the optimized un-doped Li_2_Ti_6_O_13_ structure, (**b**) CoO_6_ octahedral unit in the doped configuration, (**c**) total density of states (DOS) of Li_2_Ti_6_O_13_, (**d**) total DOS of Co-doped Li_2_Ti_6_O_13_, (**e**) atomic DOS of Co, and (**f**) constant charge density plot associated with the gap states arising from Co.

DFT simulations were carried out to look at the chemical environment of doped atoms and the electronic structures of both defect-free and doped Li_2_Ti_6_O_13_. Here, we only considered the promising dopants, as discussed earlier. In the case of Co^3+^, Co–O bond distances in the CoO_6_ unit were slightly shorter than Ti–O bond distances in the TiO_6_ unit (see Figure 8). This is because of the smaller ionic radius of Co^3+^ (0.61 Å) than that of Ti^4+^ (0.71 Å) in an octahedral coordination. The total density of states (DOS) plot indicates that Li_2_Ti_6_O_13_ is a semiconductor with a band gap of 2.90 eV. This value agrees reasonably with the estimated band gap of 3.00 eV from GGA–PBE-based DFT calculation [18] and the experimental value of 3.52 eV [18]. The doping of Co^3+^ at the Ti site introduces gap states arising from Co (3d) states, confirmed by the atomic DOS of Co (see Figure 8e) and the constant charge density plot associated with the 3d states (see Figure 8f). 

**Figure 9:**
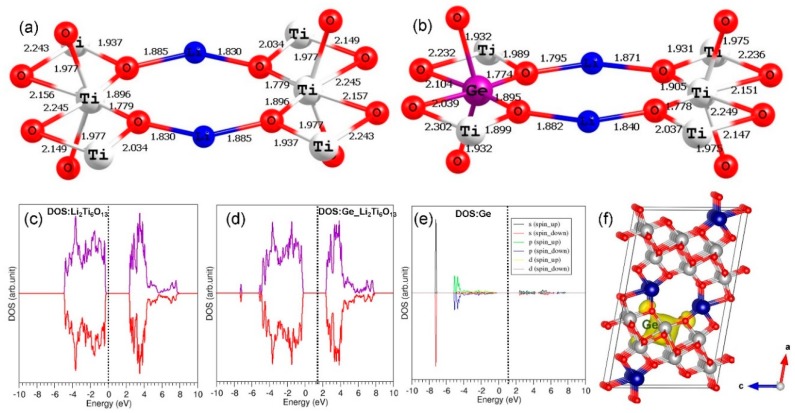
(**a**) TiO_6_ octahedral unit in the optimized un-doped Li_2_Ti_6_O_13_ structure, (**b**) GeO_6_ octahedral unit in the doped configuration, (**c**) total density of states (DOS) of Li_2_Ti_6_O_13_, (**d**) total DOS of Ge-doped Li_2_Ti_6_O_13_, (**e**) atomic DOS of Ge, and (**f**) constant charge density plot associated with the states responsible for Ge at ~−7.5 eV.

Next, we examined the relaxed configuration and electronic structure of Ge-doped Li_2_Ti_6_O_13_ and compared those with that of un-doped Li_2_Ti_6_O_13_. The Ge–O bonds were slightly shorter than the Ti–O bonds due to the smaller radius of Ge^4+^ (0.53 Å) than that of Ti^4+^ (0.71 Å) (see Figure 9). However, the degree of distortion in the bond distances was less than that observed in the case of Co^3+^. This could be due to the fact that both Ti and Ge are isovalent atoms with +4 charge. The doping of Ge dd not change the electronic structure much. The band gap is almost the same. The states arising from Ge were observed at ~−7.5 eV (deep valence band level), showing the strong bonding nature of Ge–O, and this state is confirmed by the constant charge density plot (see Figure 9f). 

## 4. Conclusions

Computational modeling techniques were applied to examine the defect energetics, Li-ion migration, solution of dopants, and electronic structures of doped Li_2_Ti_6_O_13_. Defect energetics show that the Li Frenkel was calculated to be the most stable defect process, while Schottky defects were unfavorable to occur. The Li–Ti anti-site defect was the second most favorable defect process. Li-ion diffusion took place in the *bc*-plane, with a low activation energy of 0.25 eV. The energetics of solution of trivalent dopants revealed that Li generation in the form of interstitial can be executed by doping Co^3+^ at the Ti site. Tetravalent dopant Ge^4+^ is a promising dopant at the Ti site. The efficacy of these dopants and the exact amount should be verified experimentally. Finally, doping of Co^3+^ introduced gap states, whereas Ge^4+^ did not change the complete electronic structure. In both cases, the semiconductor nature of Li_2_Ti_6_O_13_ was not altered by the both dopants. However, high concentrations of dopants are likely to influence the electronic structure of Li_2_Ti_6_O_13_.

## Figures and Tables

**Table 1:** Calculated structural parameters and corresponding experimental values reported for monoclinic (C2/m) Li_2_Ti_6_O_13_.

Parameter	Calculated		Experiment [18]	|∆| (%)	
	Force Field	DFT		Force Field	DFT
a (Å)	15.7437	15.4589	15.3065	2.82	0.99
b (Å)	3.7254	3.7719	3.7474	0.59	0.65
c (Å)	9.0525	9.2499	9.1404	0.96	1.19
α = γ (°)	90.00	90.00	90.00	0.00	0.00
β (°)	99.2636	100.03	99.3790	0.12	0.65

**Table 2:** Li–Li distances and their corresponding activation energies for the Li-ion migration in Li_2_Ti_6_O_13_ as reported in the Figure 4.

Migration Path	Li–Li Separation (Å)	Activation Energy (eV)
A	3.73	0.25
B	4.47	0.90

## Supplementary Materials

The following are available online at https://www.mdpi.com/1996-1944/12/18/2851/s1, Table S1: Interatomic potential parameters used in the atomistic simulations of Li_2_Ti_6_O_13_.
