# Supplementary material for: Defects, Diffusion, and Dopants in Li2Ti6O13: Atomistic Simulation Study
Source: Materials (Basel). 2019 Sep 4;12(18):2851. doi: 10.3390/ma12182851 (PMC6766017; doi:10.3390/ma12182851)
Supplement: Supplementary file 1 [file materials-12-02851-s001.pdf]

materials

Supplementary

Defects, Diffusion, and Dopants in $Li_2Ti_6O_{13}$: Atomistic Simulation Study

Navaratnarajah Kuganathan [1,2,*], Sashikesh Ganeshalingam [3] and Alexander Chroneos [1,2]

[1] Department of Materials, Imperial College London, London SW7 2AZ, UK
[2] Faculty of Engineering, Environment and Computing, Coventry University, Coventry CV1 5FB, UK
[3] Department of Chemistry, University of Jaffna, Sir. Pon Ramanathan Road, Thirunelvely, Jaffna 40000, Srilanka
* Correspondence: n.kuganathan@imperial.ac.uk

Received: 30 July 2019; Accepted: 3 September 2019; Published: 4 September 2019

Table S1. Interatomic potential parameters used in the atomistic simulations of $Li_2Ti_6O_{13}$.

Two-Body [$\Phi_{ij}(r_{ij}) = A_{ij} \exp(-r_{ij}/\rho_{ij}) - C_{ij}/r_{ij}^6$]					
**Interaction**	***A*/eV**	**ρ/Å**	***C*/eV·Å^6**	**Y/e**	**K/eV·Å^{-2}**
Li^+—O^{2-} [1]	632.1018	0.2906	0.00	1.00	99999
Ti^{4+}—O^{2-} [2]	5111.7	0.2625	0.000	−0.10	314.0
O^{2-}—O^{2-} [2]	12420.5	0.2215	29.07	−2.80	31.0
Al^{3+}—O^{2-} [3]	1114.9	0.2742	0.000	3.00	99999
Co^{3+}—O^{2-} [4]	1329.82	0.3087	0.000	2.04	196.30
Ga^{3+}—O^{2-} [5]	2901.12	0.2742	0.000	3.000	99999
Sc^{3+}—O^{2-} [6]	1299.4	0.3312	0.000	3.000	99999
In^{3+}—O^{2-} [7,8]	1495.65	0.3327	4.33	3.000	99999
Y^{3+}—O^{2-} [3]	1345.10	0.3491	0.000	3.000	99999
Gd^{3+}—O^{2-} [9]	1885.75	0.3399	20.34	3.000	99999
La^{3+}—O^{2-} [5]	1545.21	0.3590	0.000	−0.250	145.0
Si^{4+}—O^{2-} [10]	1315.2478	0.317759	10.141118	4.000	99999
Ge^{4+}—O^{2-} [11]	1497.3996	0.325646	16.00	4.000	99999
Mn^{4+}—O^{2-} [12]	3087.826	0.2642	0.00	4.000	99999
Sn^{4+}—O^{2-} [13]	1414.32	0.3479	13.66	4.000	99999
Ce^{4+}—O^{2-} [11]	1986.83	0.3511	20.40	7.700	291.75

References

1. Kuganathan, N.; Islam, M.S. Li_2MnSiO_4 Lithium Battery Material: Atomic-Scale Study of Defects, Lithium Mobility, and Trivalent Dopants. *Chem. Mater.* **2009**, *21*, 5196–5202.
2. Olson, C.L.; Nelson, J.; Islam, M.S. Defect Chemistry, Surface Structures, and Lithium Insertion in Anatase TiO_2. *J. Phys. Chem. B* **2006**, *110*, 9995–10001.
3. Lewis, G.V.; Catlow, C.R.A. Potential models for ionic oxides. *J. Phys. C Solid State Phys.* **1985**, *18*, 1149.
4. Cherry, M.; Islam, M.S.; Catlow, C.R.A. Oxygen Ion Migration in Perovskite-Type Oxides. *J. Solid State Chem.* **1995**, *118*, 125–132.
5. Khan, M.S.; Islam, M.S.; Bates, D.R. Dopant Substitution and Ion Migration in the $LaGaO_3$-Based Oxygen Ion Conductor. *J. Phys. Chem. B* **1998**, *102*, 3099–3104.
6. Fisher, C.A.J.; Hart Prieto, V.M.; Islam, M.S. Lithium Battery Materials $LiMPO_4$ (M = Mn, Fe, Co, and Ni): Insights into Defect Association, Transport Mechanisms, and Doping Behavior. *Chem. Mater.* **2008**, *20*, 5907–5915.
7. Minervini, L.; Zacate, M.O.; Grimes, R.W. Defect cluster formation in M_2O_3-doped CeO_2. *Solid State Ionics* **1999**, *116*, 339–349.

8. Grimes, R.W.; Busker, G.; McCoy, M.A.; Chroneos, A.; Kilner, J.A.; Chen, S.-P. The Effect of Ion Size on Solution Mechanism and Defect Cluster Geometry. *Ber. Bunsenges. Phys. Chem.* **1997**, *101*, 1204–1210.
9. Busker, G.; Chroneos, A.; Grimes, R.W.; Chen, I.-W. Solution Mechanisms for Dopant Oxides in Yttria. *J. Am. Ceramic Soc.* **1999**, *82*, 1553–1559.
10. Sastre, G.; Gale, J.D. Derivation of an Interatomic Potential for Germanium- and Silicon-Containing Zeolites and Its Application to the Study of the Structures of Octadecasil, ASU-7, and ASU-9 Materials. *Chem. Mater.* **2003**, *15*, 1788–1796.
11. Kuganathan, N.; Iyngaran, P.; Chroneos, A. Lithium diffusion in Li_5FeO_4. *Sci. Rep.* **2018**, *8*, 5832.
12. Woodley, S.M.; Catlow, C.R.A.; Piszora, P.; Stempin, K.; Wolska, E. Computer Modeling Study of the Lithium Ion Distribution in Quaternary Li–Mn–Fe–O Spinels. *J. Solid State Chem.* **2000**, *153*, 310–316.
13. Kuganathan, N.; Kordatos, A.; Chroneos, A. Li_2SnO_3 as a Cathode Material for Lithium-ion Batteries: Defects, Lithium Ion Diffusion and Dopants. *Sci. Rep.* **2018**, *8*, 12621.

© 2019 by the authors. Submitted for possible open access publication under the terms and conditions of the Creative Commons Attribution (CC BY) license (http://creativecommons.org/licenses/by/4.0/).
